# Stakeholders analysis of COVID-19 management and control: a case of Iran

**DOI:** 10.1186/s12889-022-14219-0

**Published:** 2022-10-13

**Authors:** Mohammad Mohamadian, Taha Nasiri, Mohammadkarim Bahadori, Habib Jalilian

**Affiliations:** 1 Health Management Research Center, Baqiyatallah University of Medical Sciences, Tehran, Iran; 2 Department of Health Services Management, Faculty of Health, Baqiyatallah University of Medical Sciences, Tehran, Iran; 3 Department of Health Services Management, School of Health, Ahvaz Jundishapur University of Medical Sciences, Ahvaz, Iran

**Keywords:** COVID-19, Coronavirus, COV-SARS-2, Stakeholder analysis, Policy analysis, Iran

## Abstract

**Background:**

The COVID-19 pandemic is a multi-faceted phenomenon with many political, economic and social consequences. Success in managing and controlling this pandemic depends on the coordinated efforts of many organizations and institutions. Therefore, this study aimed to identify and analyze the actors and stakeholders related to managing and controlling this pandemic in Iran.

**Methods:**

This mix-method stakeholder analysis was conducted in 2021 nationwide as retrospectively. The purposive sampling method was applied when inviting eligible participants to participate in the study. Our study was conducted in two phases. In the qualitative phase, data were collected using a semi-structured interview. An interview guide was developed based on the WHO stakeholder analysis framework. In the quantitative phase, we used a questionnaire developed based on the study framework. Each question was scored on a 5-point Likert scale, with a score greater than 4 was considered as high, 3–4 was considered as moderate, and 1–3 was considered as low. Data were analyzed using framework analysis, WHO stakeholders’ analysis framework and MENDELOW matrix. MAXQDA qualitative data analysis software Version 11 and Policy Maker software (Version. 4) were used for data analysis.

**Results:**

A total of 48 stakeholders were identified. Ministry of Health (MoH), National Headquarters for Coronavirus Control (NHCC) had the highest participation level, high supportive position, and knowledge of the subject. The Parliament of Iran (PoI), Islamic Revolutionary Guard Corps (IRGC), and Islamic Republic of Iran Broadcasting (IRIB) had the highest power/influence during the Covid-19 epidemic. Only two stakeholders (6.06%) had high participation, and 18.18% had moderate participation. All stakeholders except for the NHCC and the MoH lacked appropriate knowledge of the subject. Furthermore, only three stakeholders (9.09%) had high power/influence.

**Conclusion:**

Given the multidimensional nature of Covid-19, most institutions and organizations were involved in managing this pandemic. Stakeholders with high power/authority and resources had a low/moderate participation level and a moderate supportive position. Moreover, organizations with a high supportive position and participation had low power/authority and resources to cope with COVID-19.

**Supplementary Information:**

The online version contains supplementary material available at 10.1186/s12889-022-14219-0.

## Introduction

The COVID-19 pandemic posed unprecedented challenges to national and subnational governments and caused social and economic disruption across countries [1–6]. The severity and the spread of COVID-19 have caused governments across the world to need the participation of society and the cooperation of civil society organizations and institutions, and effective policy coordination across levels, jurisdictions, and sectors to cope with the challenges and effective management of the pandemic [2, 4, 7–9].

Coordination is an important factor in times of crisis [4, 10]. It helps avoid negative externalities, harmful competition, and intergovernmental conflict [11–17]. It can also prevent inconsistencies, fragmentation, redundancies, contradiction, and duplication [18–21]. Unlike normal situations, emergency response situations are highly complex and include high uncertainty, competing priorities, and conflicting interests among stakeholders, so collaborative planning and shared decision-making are essential [22, 23]. Since emergency management includes complex networks of tasks, resources and actors, coordination is highly important to address the embedded interdependencies for smooth and efficient response operations [24].

The effective and efficient response to the earliest stages of an outbreak requires various synergic factors related to coordinating responders, communicating risk, managing health information, and ensuring health interventions [25]. Given the multi-faceted nature of COVID-19, inter-institutional coordination, and integration, among the different organizations within health systems are required to control and manage the disease [26, 27]. Moreover, since governments' weaknesses in combating the crisis have been highlighted due to the severity, immediacy and complexity of COVID-19 [28], there is a need for all stakeholders to play their role in curbing this widespread disease [29]. One challenge that national and local governments plague in combating the pandemic is effectively coordinating actors, resources, and activities flexibly.

Given that the COVID-19 pandemic is a multi-faceted phenomenon with many political, economic and social consequences, stakeholders with different interests/power and political incentives can play an important role in handling the disease. Stakeholder analysis as a systematic tool assists policymakers in identifying, categorizing and analyzing stakeholders/ actors that can be influenced by a proposed action [30–32]. Stakeholder analysis has been established to understand the power and positions of stakeholders regarding certain new policies and assess the likely implications for the acceptability of new policies, interventions and responses [33]. Moreover, it assists managers and policymakers in finding the potential misunderstandings against a certain policy or program and adopting the necessary measures/strategies to manage it [32, 34].

The COVID-19 crisis has engaged every level of government and multiple policy sectors. Given that using global experience during a crisis is one of the most important crisis management mechanisms, a review of the strengths and weaknesses of Iran in management and control of the COVID-19 reviewing the role and status of stakeholders and actors during this time can be used as a model by other involved countries. Furthermore, given that no comprehensive study has been conducted so far in Iran to identify and analyze the role of key stakeholders and actors related to the management and control of COVID-19, this study aimed to identify and analyze the stakeholders related to the COVID-19 management and control in Iran.

## Methods

### Study design

This study is a part of a larger study entitled “Policy Analysis of COVID-19 in Iran”, which was performed using the health policy triangle framework. This study focused on the actors’ component and aimed to identify stakeholders involved in COVID-19 management and control in Iran, to provide a comprehensive analysis of their knowledge of the subject, interests, power/ influence and participation in COVID-19 policy-making. This mix-method stakeholder analysis was conducted in 2021 as retrospectively.

### Setting

This is a nationwide stakeholder analysis. All organizations and institutions that in some way played an important role in one of the stages of the COVID-19 policy-making process, including policy implementation, policy evaluation, policy agenda setting, policy formulation and policy adoption were included in our study.

### Participant selection

After identifying initial stakeholders through document analysis, key informants were recruited using a purposive sampling technique. In our study, selection criteria encompassed informants’ professional knowledge and having at least work experience in one of the stages of COVID-19 policy making, including agenda setting, policy adoption, policy formulation, policy implementation and policy evaluation or having at least one article in the field of COVID-19 management and control or crisis management and their ability to provide reflective answers to the posed questions.

### Data collection

This study was conducted in two phases. In the first phase, we identified stakeholders and their roles through the review of the policy document and reports deriving from the National Headquarters for Coronavirus Control (NHCC), Ministry of Health (MoH) [35]. We also asked the interviewees to introduce potentially other stakeholders engaged in COVID-19 management and control and to state the role of different stakeholders.

The second phase was the stakeholder analysis. This phase encompassed two parts: qualitative and quantitative. In the qualitative part, the relationships, coordination, cooperation, and conflict of interests between stakeholders, were analyzed. Data were collected using semi-structured interviews. An interview guide was developed based on the WHO stakeholder analysis framework [36]. The interview guide included interviewees' characteristics and items related to the study objectives. After developing the interview guide, this was revised and confirmed by a panel of five experts with experience in stakeholder analysis research. Also, after conducting and analyzing the first three interviews, the research team rechecked the interview guide in terms of comprehensiveness and transparency of questions. Key informants and stakeholders involved in COVID-19 management and control were interviewed face to face or via telephone in their own facilities, in a quiet private situation, during their working hours, and according to availability. The interview steps were as follows: the interview appointments were made with each participant, the interviews were scheduled at the time and place most convenient for the participant; the notes were immediately typed into one electronic following the interview; the questions were asked no more than twice; the interview was terminated at the interview's request, and the information was entered in the same words the stakeholder used. Prior to the interview, the participants were informed of the objective of the study and written informed consent was obtained from all study participants. Participants were assured all responses would remain anonymous (Supplemantry File 1).

In the quantitative part, we used a questionnaire developed based on the World Health Organization stakeholder analysis framework [36]. The questionnaire consisted of three parts: 1) an introductory section; 2) the definition of characteristics (e.g. knowledge, position, resources, influence/power, interest and level of participation); 3) and a scoring matrix including stakeholders and characteristics. The scoring of the characteristics of knowledge, position, interests, resources, power and the level of stakeholders' participation during the Covid-19 was as follows: first, each participant gave a score of 1–5 to each characteristic for each stakeholder. A higher score indicates a high knowledge of the subject, high supportive position about the policy, high interest in the subject, more resources and power to influence the subject or policy and a high level of participation in policy implementation and vice versa. The mean score was computed for each stakeholder through Excel software. A score greater than 4 was considered as high, 3–4 was considered as moderate, and 1–3 was considered as low. After completing the three questionnaires, the reliability of the questionnaire was assessed through cross-reference, and the mean scores of the characteristics for five stakeholders were compared with the interviewees' statements about the characteristics of each stakeholder (Supplemantry file 2).

### Data analysis

Data were analyzed using framework analysis, WHO stakeholders’ analysis framework and MENDELOW matrix. MAXQDA qualitative data analysis software Version 11 and Policy Maker software (Version. 4) were used for data analysis.

### Trustworthiness

To ensure trustworthiness, the researchers put aside their political orientation and served impartially at all stages. Heterogeneous participants were interviewed, and triangulation in data sources, including key informants, relevant documents, and stakeholders' websites, was performed. Moreover, the researchers' interpretations of interviews were given again to interviewees and rechecked by them to avoid misinterpretation.

## Results

### Stakeholders identification: key actors and stakeholders of COVID-19 management and control in Iran

A total of 36 participants were recruited to interview. The interviewees consisted of twelve mid-level state managers, nine top governmental managers, three NGO members, five health professionals and lawyers, three specialists in infectious disease, two researchers, one country director of international organizations and one legislator (Member of Parliament) were interviewed. The length of the interviews ranged from 60 to 70 min. Interviewees included 25 men and 11 women. The mean age of participants was 48 years of age, ranging from 35 to 64 years old. The mean work experience of participants was 14 years and ranged from 10 to 31 years.

Given that the COVID-19 pandemic is a multi-faceted phenomenon with wide dimensions, the majority of economic, cultural, social, political and executive organizations and institutions can play a crucial role in handling the disease.

A total of 48 stakeholders related to the COVID-19 management and control were identified. Tables 1 and 2 represent the stakeholders involved in COVID-19 in Iran and their present/active role. Their roles are divided into seven main categories: policy-making, executive, coordinative, supportive, monitoring/supervision, advisory, and informing. 14 ministries out of 19 in the country were among stakeholders and played a role during the pandemic. As one of the key stakeholders, MoH played a major role in each of the seven categories and was more engaged during the pandemic. Three stakeholders (9.09%) (The Parliament of Iran (PoI), Ministry of Economic Affairs and Finance (MEAF), NHCC and MoH) had a major role in policy-making. Most of the organizations had executive roles (≈ 70%), supervision roles (≈ 58%) and supportive roles (≈ 40%). Six stakeholders (9.09%) had a coordinative role. Only MoH had an advisory role. Four stakeholders (≈ 12%) had informing role during the pandemic.

**Table 1:** Present/active stakeholders and their roles in COVID-19 management and control in Iran

Agencies/ Organizations	Role
**The Parliament of Iran (PoI)**	•Monitoring the performance of the government and other organizations and stakeholders in handling the COVID-19 •Laying down laws and regulations related to compliance with health protocols and standards •Laying down supportive economic laws
**National Headquarters for Coronavirus Control (NHCC)**	•Policy-making and planning •Facilitate and synergy in inter-sectorial cooperation •Mobilizing all capacities to import foreign vaccines and produce domestic vaccines for public vaccination, especially for vulnerable groups (identified by the NHCC) •Identifying problems and providing solutions in the form of executive approvals •Synergy and strengthening of various capabilities across the country, directing and Mobilizing all capacities within the country, including public and private, military and law enforcement forces and non-governmental organizations, mobilization, religious organizations, mosques and charities
**Ministry of Health**	•NHCC advisory role and providing valid information and evidence to NHCC •Implementation of preventive, diagnosis and treatment measures •Informing and public education •Allow re-export of surplus imported pharmaceutical and consumer items •Formulation of clinical guidelines and preventive protocols •Monitoring the proper implementation of interventions •Coordination in informing •Official information reference in the field of Covid-19 management and control •Formulation and implementation of equitable distribution of Covid-19 vaccines and medicines •Purchase of COVID-19 medicines, supplies, and vaccines) •Receive cash assistance to cope with the COVID-19 in coordination with the Central Bank •Holding employment tests in accordance with health instructions •Necessary training for the Red Crescent population to monitor the process of actions •Import of COVID-19 vaccine •Monitoring the process of actions of the Red Crescent Society •Training and consulting with other related organizations and institutions
**Islamic Republic of Iran Army**	•Use of army health and medical capacities •Cooperation in controlling entry and exit from the inside and outside the country
**Islamic Revolutionary Guard Corps/ Basij Armed force**	•Using the health and medical capacities of the IRGC •Cooperation in controlling entry and exit from the inside and outside the country •Cooperation in informing and persuading people •Cooperation in identifying, following up, isolating and quarantining infected patients •Supporting the poor and needy patients •Participation in the gathering and marches
**Disciplinary Command of the Islamic Republic of Iran**	•Using the health and medical capacities of the Disciplinary Forces •Cooperation in controlling entry and exit from the inside and outside the country •Dealing with violators in the field of compliance with protocols and health standards •Supervising public places and food preparation and distribution centers •Supervising the holding of social, cultural and religious ceremonies
**Iranian Traffic Police**	•Enforcement of rules of prohibition of road traffic
**Red Crescent**	•Participation in service delivery (prevention and vaccination services) •Participation in cross-border traffic control •Participation in the provision of the workforce, physical space, equipment and supplies •Cooperation in informing and persuading people
**Ministry of Roads and City Planning**	•Supporting businesses affected by COVID-19 •Providing facilities for air and rail transport •Monitoring the housing market price relations (supporting tenants) •Participation in the implementation of the ban on inland traffic •Creating a database of patients and tracking
**Islamic Republic of Iran Broadcasting**	•Continuous information and education, and community awareness •Free advertising to develop online businesses and motivate online sales
**Information Committee of National Headquarters for Coronavirus Control**	•Continuous information and training •Planning and implementation of persuasive plans to encourage the public to use the facemask continuously indoor places
**Social-Security Committee**	•Delegating provincial powers •Holding mourning ceremonies •Coordination in information •Information reference in the areas of restrictions or closures of jobs at the national and provincial levels •Monitoring crimes and how the law is enforced •Closing down union units and places in the high-risk group
**Municipality**	•Participation in the implementation of curfew interventions •Closure of public, commercial and entertainment centers
**Governor/ governor-general**	•Provincial information Source •Monitoring the implementation of health protocols and law enforcement
**Hajj and Pilgrimage Organization**	•Implementation of decisions related to Hajj and pilgrimage
**Planning and Budget Organization**	•Support measures for businesses affected by COVID-19 •Funding COVID-19 management •Purchasing COVID-19-related health items •The necessary financing to purchase corona-related health items •Financial support and budget allocation
**Ministry of Culture and Islamic Guidance**	•Identifying regulatory departments to monitor National Staff approvals •Participation in the implementation of the approvals of the NHCC •Monitoring the activities of integrated cultural and social centers
**Ministry of Cultural Heritage, Handicrafts and Tourism**	•Participation in the implementation of the decisions of NHCC
**Ministry of Education**	•Participation in the implementation of the approvals of the NHCC •School closures •Creating a virtual learning platform •Monitoring students' cultural and social gatherings
**Ministry of Science, Research and Technology**	•Participation in the implementation of the approvals of the NHCC •Closure of universities and higher education centers •Creating a virtual learning platform •Monitoring students' cultural and social gatherings
**Ministry of Economic Affairs and Finance**	•Issuing licenses for import and export of drugs and consumables •Granting facilities for air and rail transportation •Economic support for affected businesses and guilds •Participation in the implementation of the approvals of the NHCC •Facilitate and develop electronic processes
**Ministry of Cooperatives, Labour, and Social Welfare**	•Supporting more affected business units •Supporting more affected households •Dealing with the economic consequences resulting from the COVID-19 outbreak
**Organization of Agriculture**	•Implementing interventions to ensure food security during the epidemic
**Ministry of Foreign Affairs**	•Efforts to attract the support of international organizations •Customs exemption for gifts and aid related to COVID-19 •Cooperation in cross-border relations regarding disease control
**Ministry of Interior**	•Monitoring the implementation of all interventions (monitoring the implementation of health instructions and protocols) •Participation in the implementation of the approvals of the NHCC •Monitoring all collective social, cultural, commercial, recreational, political and religious activities •Implementation of traffic plan in Tehran •Identifying regulatory departments to monitor National Staff approvals •Tracing the mutated virus in the world and neighbouring countries
**Ministry of Industry, Mine & Trade**	•Participation in the implementation of the approvals of the NHCC •Identifying oversight departments to oversee National Staff approvals •Support for the most affected business units from COVID-19 •Import and export of pharmaceutical and consumer items related to COVID-19 •Implementation of traffic plan in Tehran •Monitoring the activities of aggregate business units •Dealing with the economic consequences of the COVID-19 outbreak
**Ministry of Information and Communications Technology of Iran**	•Evaluating the observance of health instructions •Creating a database of patients and tracking •Participation in identifying and tracking people with a positive test •Development of e-government to reduce face-to-face visits
**Ministry of Sport and Youth**	•Using the capacity of sports venues for vaccination •Information and training •Monitoring the closure and reopening of indoor sports facilities
**Ministry of Intelligence**	•Tracing of mutant virus in the world and neighbouring countries
**Public transportation system (air, land and sea)**	•Participation in the implementation of the approvals of the NHCC •Strengthening the inner town and suburban public transportation fleet
**Central Bank of the Islamic Republic of Iran**	•Facilities payment to more affected business units than Corona •Relocation of unused resources to compensate for household livelihoods •Dealing with the economic consequences of the corona outbreak •Participation in providing the funds needed to implement the approvals of the NHCC •Extension of facilities for public transport
**National Development Fund of Iran**	•Financing for a part of the cost of managing the COVID-19 epidemic, especially in the field of medicine and medical equipment
**Social Security Organization**	•Helping control the spread of the disease and break the virus transmission chain •Services to patients with COVID-19 •Expanding basic insurance obligations in indirect treatment •Supporting businesses, manufacturing companies and employers •Supporting workers and the insured •Educational, cultural and research measures against COVID-19 •Payment for unemployment days during the outbreak of COVID-19 disease from the resources of the National Development Fund
**Organization for Combating Smuggling of Goods and Currency (Medication & Vaccine)**	•Necessary measures to prevent illegal withdrawal of drugs and vaccines
**Judiciary**	•Guarantee the implementation of the resolution of the NHCC •Lease extension agreements •Leave extension for non-dangerous prisoners
**Justice**	•Guarantee the implementation of the resolution of the NHCC
**Dispute Resolution Council**	•Guarantee the implementation of the resolution of the NHCC regarding the lease extension agreement
**Attorney General**	•Dealing with violators •Supporting the implementation of protocols
**Trustees of the Holy Astans**	•Participation in the implementation of the approvals of the NHCC •Closing and reopening of Holy Astans
**Friday Prayer Policy Council**	•Monitoring the holding of congregational prayers by observing health protocols
**Islamic Development Coordination Council**	•Participation in holding Quds Day ceremonies (marches) in low-risk cities
**General Staff of the Armed Forces**	•Monitoring cultural and social gatherings •Providing a part of the required personnel of the MoH •Smart epidemic management platform (Omid system)
**Executive agencies at the national and provincial levels**	•Participation in the implementation of health protocols •Prohibition of holding gatherings contrary to health regulations •The provision of health and livelihood programs during COVID-19 •Monitoring the implementation of the approvals of the NHCC at all administrative and social levels and law enforcement
**Pharmaceutical companies**	•Providing essential medicine for COVID-19 patients
**drugs and medical equipment importing companies**	•Importing medicine and medical equipment related to the patient's treatment
**Vaccine development companies**	•Investment and research in vaccine development
**Basic and Complementary insurance**	•Financing the provision of services to patients infected with COVID-19
**Private hospitals**	•Providing services to patients infected with COVID-19

**Table 2:**
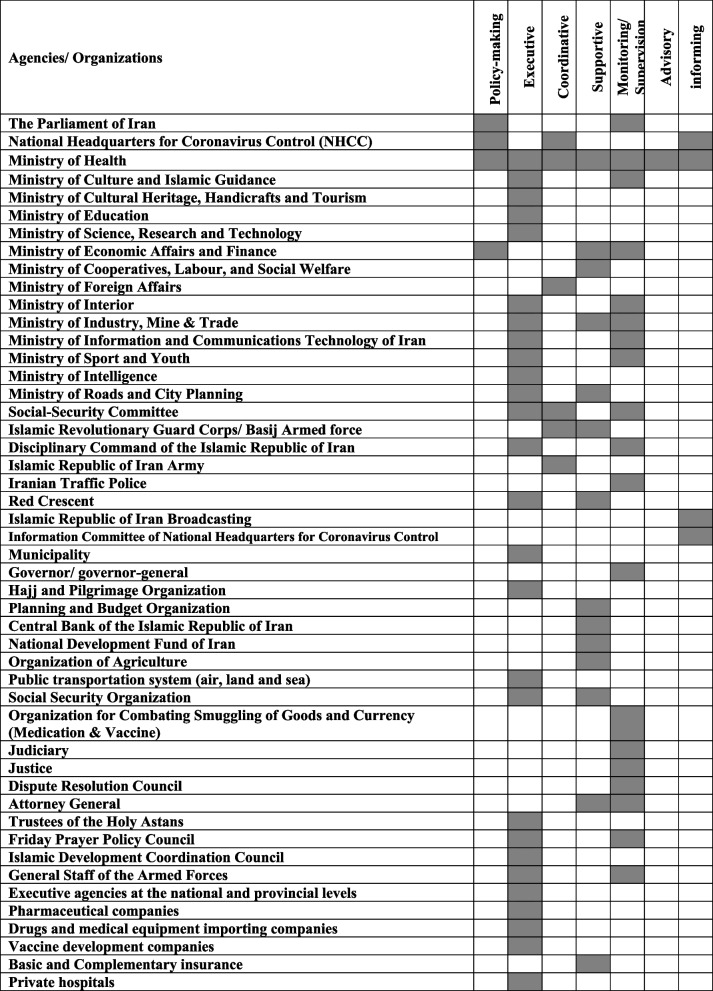
Classification of stakeholders by their role in the management and control of Covid-19

### Characteristics of COVID-19 stakeholders

Table 3 shows the characteristics of the main stakeholders of COVID-19 management and control in Iran. Since COVID-19 was a novel virus/ pandemic, most stakeholders' level of knowledge was low. All stakeholders except for the NHCC and MoH lacked enough knowledge of the subject.

**Table 3:** Status of knowledge, position, interests, resources, power and level of stakeholders' participation during the Covid-19

Stakeholders	Knowledge of the subject	Position	Interests	Resources	Power/influence	Level of participation
The Parliament of Iran	Low	Moderate supportive	Low	Moderate	High	Low
National Headquarters for Coronavirus Control	High	High supportive	High	Moderate	Moderate	High
Ministry of Health	High	High supportive	High	Moderate	Moderate	High
Islamic Republic of Iran Army	Low	Low supportive	Low	Low	Low	Low
Islamic Revolutionary Guard Corps	Low	Moderate supportive	Moderate	Moderate	High	Moderate
Disciplinary Command of the Islamic Republic of Iran	Low	Low supportive	Low	Low	Low	Low
Iranian Traffic Police	Low	Moderate supportive	Low	Low	Low	Moderate
Islamic Republic of Iran Broadcasting	Low	Moderate supportive	Moderate	Moderate	High	Moderate
Municipality	Low	Low supportive	Low	Low	Moderate	Low
Governor/ governor-general	Low	Moderate supportive	Moderate	Moderate	Moderate	Moderate
Hajj and Pilgrimage Organization	Low	Low supportive	Low	Low	Low	Low
Planning and Budget Organization	Low	Low supportive	Moderate	Moderate	Moderate	Low
Ministry of Culture and Islamic Guidance	Low	Low supportive	Low	Low	Low	Low
Ministry of Cultural Heritage, Handicrafts and Tourism	Low	Low supportive	Low	Low	Low	Low
Ministry of Education	Low	Low supportive	Moderate	Low	Low	Low
Ministry of Science, Research and Technology	Low	Moderate supportive	Moderate	Low	Low	Moderate
Ministry of Economic Affairs and Finance	Low	Low supportive	Moderate	Moderate	Moderate	Low
Ministry of Cooperatives, Labour, and Social Welfare	Low	Low supportive	Low	Moderate	Low	Low
Organization of Agriculture	Low	Low supportive	Low	Low	Low	Low
Ministry of Interior	Low	Moderate supportive	Moderate	Moderate	Moderate	Moderate
Ministry of Industry, Mine & Trade	Low	Low supportive	Low	Low	Low	Low
Ministry of Information and Communications Technology of Iran	Low	Low supportive	Moderate	Moderate	Moderate	Low
Ministry of Sport and Youth	Low	Low supportive	Low	Low	Low	Low
Public transportation system (air, land and sea)	Low	Low supportive	Moderate	Low	Low	Low
Central Bank of the Islamic Republic of Iran	Low	Low supportive	Moderate	Moderate	Moderate	Low
National Development Fund of Iran	Low	Low supportive	Moderate	Moderate	Moderate	Low
Social Security Organization	Low	Moderate supportive	Moderate	Moderate	Low	Low
Organization for Combating Smuggling of Goods and Currency (Medication & Vaccine)	Low	Low supportive	Low	Low	Low	Low
Judiciary	Low	Low supportive	Low	Moderate	Moderate	Low
Trustees of the Holy Astans	Low	Low supportive	Low	Low	Low	Low
Friday Prayer Policy Council	Low	Low supportive	Low	Low	Low	Low
Islamic Development Coordination Council	Low	Low supportive	Low	Low	Low	Low
Pharmaceutical companies	Low	Low supportive	Moderate	Moderate	Moderate	Low
Drugs and medical equipment importing companies	Low	Low supportive	Moderate	Moderate	Moderate	Moderate
Vaccine development companies	High	Low supportive	High	High	High	Moderate
Basic and Complementary insurance	Low	Moderate supportive	High	Moderate	Moderate	Moderate
Private hospitals	Low	Moderate supportive	Moderate	Moderate	Moderate	Moderate

The participation of all stakeholders except for the National Headquarters for Coronavirus Control (NHCC), Ministry of Health (MoH), and vaccine development companies was low/moderate. Only two stakeholders (MoH and NHCC) had a high level of participation. Four stakeholders (MoH and NHCC, vaccine development companies, and basic and complimentary insurance) were highly interested in the COVID-19 management and control. Although MoH, NHCC and vaccine development companies had a high level of knowledge and interest, they had moderate power/influence to implement interventions and decision-making. All stakeholders except for vaccine development companies had low/moderate resources, indicating the country faced a shortage of resources. The Parliament of Iran (PoI), Islamic Revolutionary Guard Corps (IRGC), and IRIB had low participation in COVID-19 management and control in the country despite their high power/influence.

One respondent pointed out that *“The MoH have not enough authority. The highest official of the country must take responsibility (i.e. the president or vice president), the MoH alone cannot, as in terms of the organization is even weaker than some ministers.”*

Moreover, most active stakeholders in COVID-19 management and control had a low supportive position to cope with COVID-19, except for NHCC and MoH, which had a high supportive position. MoH and NHCC directly deal with COVID-19 patients and strongly support COVID-19 programs. However, they had moderate resources and power/influence. Eight stakeholders (the Parliament of Iran, Islamic Revolutionary Guard Corps, Iranian Traffic Police, Islamic Republic of Iran Broadcasting, Governor/ governor-general, Social Security Organization, Basic and Complementary insurance and Private hospitals) had moderate supportive and supported the subject slightly. Most of the stakeholders had a low supportive position and low participation in this area because they did not have direct responsibility or duty to fight against COVID-19. While vaccine development companies had high knowledge of the subject, and high interest, resources and power/influence, they had low supportive positions and participation.

Influence/power is defined according to the number of resource availability and the level of power utilization by stakeholders, potential capacity and resources, including money, authority, political power, knowledge etc., to influence policy decisions. Our finding showed that only four stakeholders (the Parliament of Iran, Islamic Revolutionary Guard Corps, Iranian Traffic Police, Islamic Republic of Iran Broadcasting and vaccine development companies) had a high power to cope with the disease. Fifteen stakeholders had moderate power, and the remaining had low power. While the Parliament of Iran (PoI), Islamic Revolutionary Guard Corps (IRGC), and Islamic Republic of Iran Broadcasting (IRIB) had the highest power/influence in controlling and managing COVID-19, they had low knowledge of the subject. These stakeholders also had low/moderate participation and interest in involving the pandemic.

**Fig. 1:**
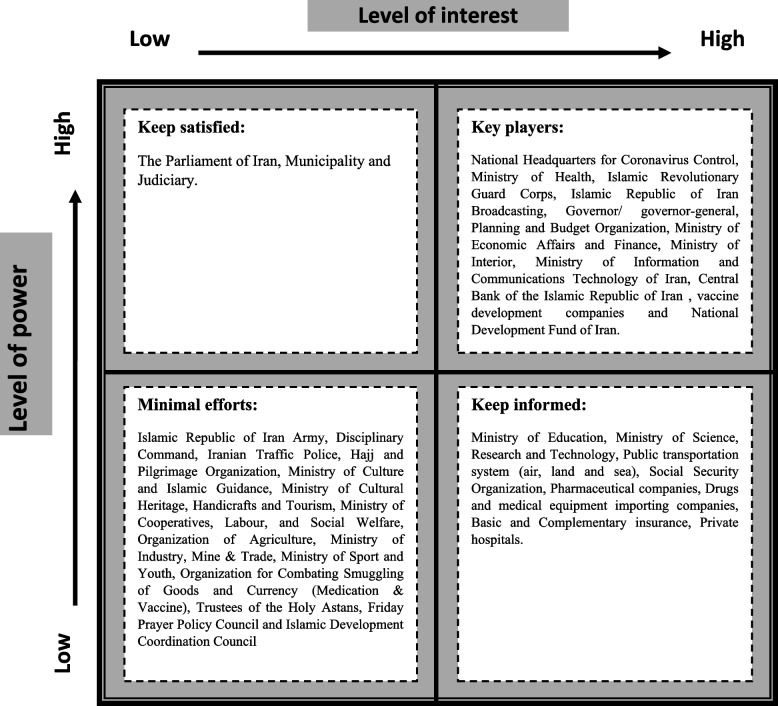
Mendelow matrix, the position of stakeholders, based on their level of power and interests

Figure 1 indicates stakeholders' level of interest and power related to the COVID-19 management and control. This matrix will be the basis for the compilation of necessary strategies to manage stakeholders in COVID-19. NHCC, MoH, IRGC, R&D, MoI, governor and governor-general, Program and Budget Organization (PBO), Ministry of Economy and Finance (MoEF), Ministry of Communications and Information Technology (MoCIT), CBIR and National Development Fund (NDF) were the main COVID-19 control-related stakeholders and had the highest involvement in the subject and highest power. Therefore, stakeholders must engage actively in policy-making and planning of the pandemic's management and control.

A participant explained: “*The unscientific interventions of decision-making bodies in the COVID-19 control and management have led to fragmentation and, ultimately, to the ineffectiveness of pandemic control and containment policies*”.

The MoEF, the PBO, the CBIR, and the NDF greatly influenced the implementation of policies and interventions by controlling financial resources. As the main body for maintaining and promoting community health and having financial, human and physical resources, the MoH was involved in all stages of policy-making, planning and implementation of interventions (prevention, control and treatment). In addition, the MoH was the main source of information in this regard.

Participants noted: “*In my opinion, the policy-making process and the content of COVID-19 control policies are incorrect and require coherent and integrated management. The correct information is provided only through the MoH and gaining public trust. However, we also see that MoH and the scientific community provide false information*”.

The Parliament of Iran was not sufficiently involved with the pandemic, despite its strong influence through the instituting of binding laws on other institutions and organizations. Moreover, although the MoE, Ministry of Science, Research and Technology (MSRT), the Social Security Organization (SSO) and the Travel Transport Organization (TTO) (air, land and sea) were among the main actors, they did not have enough influence. In the policy formulation stage, it is necessary to consult with these organizations, as for the successful implementation of policies, these organizations’ active cooperation and participation are essential.

## Discussion

In this study, an attempt was made to identify and analyze the role of stakeholders in response to the COVID-19 in Iran. Due to the multi-faceted nature of Covid-19, there is a need for wide inter-sectoral collaboration and communication among various stakeholders. Although Covid-19 is a health problem, it has broad socio-economic, cultural and political dimensions. Therefore, the MoH is not able to manage this phenomenon alone and needs the active role and coordination/collaboration of other economic and social institutions.

Our results showed that, despite the high interest, high supportive position and high level of participation in COVID-19 management and control, MoH, as a key body, did not have enough power and authority to influence other stakeholders and mobilise and mobilize and coordinate the efforts and interventions of other stakeholders. The government must reinforce and strengthen the role and position of the MoH as the main body of crisis management, especially in the field of health crises. In this case, the MoH can lead and coordinate in addition to its executive roles, such as preventing, detecting and treating infected people. Early on, in Iran, the President and the regime authorities usually opposed the imposition of strict measures and some basic health protocols such as quarantine or nationwide curfews to mitigate COVID-19 [37–39]. In addition, the Iranian MoH was pressured to lift the existing measures against the COVID-19 outbreak [37]. Previous studies in Indonesia demonstrated that the weak role of the MoH in decision-making and overlapping authority (parallelism and multiplicity) was one of the most important challenges in managing and controlling Covid-19 [40, 41]. By contrast. Sri Lanka was one of the countries that could successfully combat the pandemic. MoH had the authority to enforce the laws entrusted to them by the quarantine and prevention of disease ordinance to make provisions for preventing the spreading of infectious or contagious diseases from protecting the community [42].

Inter-governmental and state-society relationships can shape the coordination of subnational policy responses to COVID-19 in countries with different political systems [43]. A study examined intergovernmental coordination and responses in three different countries (Australia, Canada, Germany, and Switzerland) during the COVID-19 and suggested that the strength of intergovernmental councils in normal times had little effect on the extent of intergovernmental coordination during a crisis [44]. In contrast, a study showed that intergovernmental coordination practices by implementing nationwide policies and exerting a strong national leadership in Germany, Denmark and Australia led to their success in controlling the first wave of Covid-19 [1].

Agustino and Wicaksana (2020) conducted a qualitative study to analyse the efforts of the governments of China, South Korea, Italy, and Indonesia to COVID-19 response. They showed that in all four countries, various actors were actively involved in resolving a pandemic, starting with the country's highest leadership, medical staff and nurses, security agencies, researchers, and so on. In all countries, the government were the main backbone in the formulation of the policy process, which many stakeholders then implemented to control and manage the spread of the disease [45]. Similarly, the previous study on the epidemics of SARS and MERS has demonstrated that governments and healthcare systems can overcome outbreaks on a vast and sustainable level on the condition of readiness and responsiveness on a vast and sustainable level [46]. Amaratunga examined how the crisis was managed and governed in Sri Lanka. They showed that the government emulated a multi-sectoral approach to cope with the pandemic with the involvement of diverse stakeholders ranging from various institutions under the purview of the MoH and Indigenous Medical Services, the military, police, and sub-national level government officers to the private sector [47]. Previous studies also revealed that coordination between national and subnational governments in crisis responses is highly important [43, 48].

Due to the intense political and party competition and conflict of interest between some institutions and organizations, which led to inconsistencies and prevented integrated and coordinated measures, MoH failed to decide and implement interventions properly. Conflicts of interest and political and partisan competitions can lead to the adoption and implementation of uncoordinated and conflicting policies and interventions. Political conflict, such as disagreements on appropriate policy or outright power struggles [49], also hinders cooperation. A study in Indonesia found many conflicts of interest in formulating and implementing public policy handling COVID-19. Different groups and actors with different and conflicting interests affected the decision-making process and the adoption and implementation of policies [41].

We found that, despite the NHCC and the role of the MoH as the main body of health, there were numerous decision-making and policy-making centers, and in many cases, actions were taken in a non-integrated, uncoordinated and parallel manner, which, in turn, led to a reduction in the effectiveness of interventions and underuse of available resources optimally. Because there was no integrated and cohesive crisis management structure in the country to coordinate the efforts of all organizations and institutions. NHCC was created in response to coordinating an integrated leadership for combating the pandemic. This organization acted as the main policy maker council, and most of the organizations/institutions and presidents were the main members of it. This organization, like the MoH, had high Knowledge, high supportive position and interest and a high level of participation on the subject, but it had moderate resources and power/influence and had no executive power and authority. Experiences of different countries indicated that organizational structures for crisis management, clear demarcation of roles, coordination and integration between departments and ministries, and a high level of early cooperation among all stakeholders facilitated the management and control of Covid-19.

Moreover, Vietnam, a country with under-resourced, acted well because of very good communication and a high level of early cooperation among all stakeholders [50, 51]. In Colombia, structural impediments to coordination and the importance of motivational or ‘soft’ factors for supporting coordination were identified as factors influencing the coordination between institutions [52]. Timely communication on any developments of the outbreak from the government and the media, combined with up-to-date research on the new virus by the Vietnamese science community, have altogether provided reliable sources of information [51].

A study compared France, Belgium, and Canada's policy responses to the COVID-19 pandemic and showed that Belgium was the most affected by Covid-19 because the shared responsibility between national and regional governments has slowed down decision-making processes or even caused policy choices that reflected compromises between stakeholders and thus was not optimally effective [53].

The pandemic worsened the situation because the country had already been targeted under sanctions and faced instability, recession, and inflation. This reduced the possibility of many control interventions, such as quarantine and closure. Our results demonstrated that the Budget and Planning Organization and Ministry of Economic Affairs and Finance, as the two main actors had a low supportive position and low participation during the pandemic. The insufficient support from these stakeholders caused the key bodies such as MoH and NHCC not to have sufficient resources to combat the disease and implement holistic interventions. In addition, they did not have sufficient resources to provide financial and economic support to the low-income and vulnerable groups, leading to the inefficiency of some interventions and increased social dissatisfaction. In other countries like South Asia and Malaysia [54–56], economic fluctuations, poverty-inequality, and devaluation of the currency aggravated pressure on medical resources brought on by COVID-19. Chen et al. Examined the associated factors that affect the transmission of Covid-19 in six countries (China, Korea, Japan, Italy, the United States, and Brazil) and found that individuals with low socioeconomic status were more susceptible to contracting the disease and have a higher probability of dying due to an insufficient supply of medical services. Medical and socioeconomic inequality may have multiple effects on the spread and mortality of Covid-19 [57].

In Iran, conflict of interest between different groups in different areas of management and control of Covid-19, especially in the field of medicine and vaccine imports, was another factor that led to ineffective management of the disease. We found that vaccine development companies were among the stakeholders with high knowledge of the subject and high interest, resources and power/influence, but they had low supportive positions and participation. Furthermore, pharmaceutical companies and importation of medicine and medical equipment importing companies had a low supportive position. This can be attributed to their personal interests and their preferences for national vaccines inhibiting the timely import of vaccines. Conflict of interest stems from structural problems and weak rules and regulations. The high influence of some individuals/institutions in the country's political structure had led to a conflict of interest over the profitability of selling related medicines instead of valid vaccination. This led to disruption of timely vaccine imports, delays in vaccination, and an increase in the prevalence and mortality of COVID-19. Lack of transparency in the role and duties of various individuals and institutions and the weakening of the role of the MoH in crisis management exacerbated this. Moreover, the presence of many decision-making centers and the lack of unity of command caused the MoH not to have sufficient political and executive powers and, in many cases, could not implement the policies well.

Hospitals, as one of the main actors, faced many problems, including a shortage of medicine, personal protective equipment (PPE), and beds. Although they had a very high level of participation and interest, they confronted a lack of PPE and human resources, which challenged them in fulfilling their role. Laboe et al. demonstrated that owing to COVID-19, hospital admissions increased worldwide and put health care workers (HCWs) under more stress and strain, leading to increased suicide among HCWs and physicians [58]. Another study demonstrated that the prevalence of mental disorders and burnout were significantly higher among the women HCWs compared to the men, and mental disorders and burnout were higher among healthcare workers in hospitals than those working in primary healthcare centres [59].

Moreover, one of the reasons that led to the high mortality counts and the spread of the virus was the disinformation of Islamic Republic of Iran Broadcasting. Poor monitoring of mass media and cyberspace led to rumours, disinformation and false/fake information about the disease, causing fear and confusion among the people. Our results showed that although the Islamic Republic of Iran Broadcasting had low knowledge of the subject, this had high power/influence, moderate supportive position, interest and resources. Lack of integration and coordination in terms of data transparency also in Indonesia was considered as a major obstacle to success in controlling the pandemic [40]. There is no doubt that during epidemics, media can play a pivotal role in people's mental health, especially those affected by the virus. To stem the spread of the virus, people must stay at home due to some restrictions and feel depressed and anxious. Hence, the media can positively influence people's mental health by displaying entertaining programs. From the mental health perspective, the COVID-19 pandemic affected the mental well-being of individuals, as due to its restrictions, individuals lived in isolation and quarantine, which negatively affected the mental health of the population worldwide [60]. Previous studies also demonstrated that COVID-19 led to an increase in the symptoms of various psychiatric disorders such as depression, anxiety, panic disorder, physical health symptoms like pain, obsessive–compulsive disorder (OCD), and post-traumatic stress disorder (PTSD) [61–63].

### Conclusions and policy implications

Given the multidimensional nature of Covid-19, most institutions and organizations were involved in managing this pandemic. Stakeholders with high power/authority and resources had a low/moderate participation level and a moderate supportive position. Moreover, organizations with a high supportive position and participation had low power/authority and resources to cope with COVID-19. Due to the lack of a long-term and strategic approach to crisis management, there was no holistic strategic crisis plan for crisis management in the country. Failure to respond in a timely and consistent/coordinated manner to the NHCC led to negligence in implementing policies, interventions and protocols set by individuals and organizations. The lack of necessary support for the proposals/comments/opinions of the MoH in the NHCC and the lack of executive guarantees from the highest levels of government to implement health decrees, regulations and protocols caused a lack of rapid implementation of laws.

Moreover, due to the lack of transparency in the role of each organization/institution in crisis management and control, the measures and strategies/interventions taken were uncoordinated. Some strategies are required for the appropriate management of COVID-19 potential stakeholders. The necessary measures should take place to remove managerial, financial, and facilities obstacles regarding active actors/stakeholders. In the case of less active actors, apart from the requirement to increase their political and legal support, they should be encouraged to have more participation during the pandemics. Regarding inactive actors/stakeholders, it is necessary to transform their neutral position into a supportive position by designing a suitable training package and engaging them actively in playing a more active and direct role by offering appropriate informational and financial incentives. Further research is still needed to impact COVID-19 further and the role of key stakeholders/ actors in managing crises.

## Supplementary Information

**Additional file 1: Supplemantry File 1.** Interview guide. This file includes the interviewees' characteristics and questions related to stakeholders analysis.

**Additional file 2: Supplemantry File 2.** Stakeholders analysis checklist. This file includes the status of knowledge, position, interests, resources, power and level of stakeholders' participation during the Covid-19.

## Data Availability

Data will be available upon reasonable request from the corresponding author.
