# Supplementary material for: Stakeholders analysis of COVID-19 management and control: a case of Iran
Source: BMC Public Health. 2022 Oct 13;22:1909. doi: 10.1186/s12889-022-14219-0 (PMC9559115; doi:10.1186/s12889-022-14219-0)
Supplement: Supplementary file 1 — Additional file 1: Supplemantry File 1. Interview guide. This file includes the interviewees' characteristics and questions related to stakeholders analysis. [file 12889_2022_14219_MOESM1_ESM.doc]

Supplemantry File 1: Interview guide. This file includes the interviewees' characteristics and questions related to stakeholders analysis.

Interviewee code:

Position and organization:

Age:

Gender:

Work experience:

Interview Date:

1- In your opinion, who are the stakeholders/ actors (i.e. individuals, organizations, and government) and the influential stakeholders of COVID-19 policymaking and can play an important role in managing and controlling COVID-19?

2- In your opinion, which institutions and organizations influence the process of COVID-19 policymaking?

3- In your opinion, which institutions or organizations have been influenced by the COVID-19 pandemic?

4- In your opinion, what role does each relevant institution or organization play in the management and control of COVID-19?

5- How is participation and cooperation between different stakeholders (institutions and organizations involved in managing and controlling COVID-19)?

6- In your opinion, is there a conflict of interest between involved institutions and organizations in controlling and managing COVID-19? If yes, between which organizations and how?
