# Supplementary material for: Stakeholders analysis of COVID-19 management and control: a case of Iran
Source: BMC Public Health. 2022 Oct 13;22:1909. doi: 10.1186/s12889-022-14219-0 (PMC9559115; doi:10.1186/s12889-022-14219-0)
Supplement: Supplementary file 2 — Additional file 2: Supplemantry File 2. Stakeholders analysis checklist. This file includes the status of knowledge, position, interests, resources, power and level of stakeholders' participation during the Covid-19. [file 12889_2022_14219_MOESM2_ESM.doc]

Supplemantry File 2: Stakeholders analysis checklist. This file includes the status of knowledge, position, interests, resources, power and level of stakeholders' participation during the Covid-19.

ID number:

Interview Date:

Position and organization:

Dear participant

- This questionnaire has been developed to analyze the stakeholders related to managing and controlling the COVID-19 pandemic in Iran.
- The list of stakeholders/actors has already been identified and extracted through document review.
- Management and control of COVID-19 refer to all stages of the policy-making process, including policy agenda setting, policy formulation, policy adoption, policy implementation, and policy evaluation.
- Please score ranging from 1 to 5 to each stakeholder regarding their knowledge of the subject, position, interests, resources, power, influence, and level of participation in the subject. A higher score indicates a high knowledge of the subject, high supportive position about the policy, high interest in the subject, more resources and power to influence the subject or policy and a high level of participation in policy implementation and vice versa.
- The information obtained through these interviews will only be used for the study objectives. Be assured that all responses will remain anonymous.
- Please put aside your political orientation and observe impartially at all stages.
- “Knowledge” of the subject is defined as the level of accurate knowledge the stakeholder has on the policy under analysis and how each stakeholder defines the policy in question.
- “Position” is defined as follows: A) the interaction with other actors involved in the policy; and whether the stakeholder supports, opposes, or is neutral about the policy, which is key to establishing whether or not he/she will block the policy implementation; B) stakeholder’s status as a supporter or opponent of the policy.
- “Interest” is defined as the extent of involvement in the policy and the stakeholder’s interest in the policy, or the advantages and disadvantages that implementation of the policy may bring to the stakeholder or his/her organization.
- “Resources” are defined as the number of resources—human, financial, technological, political, and other—available to the stakeholder and his/ her ability to mobilize them. A power index also summarises this characteristic and determines the level of force with which the stakeholder might support or oppose the policy.
- “Influence/power” is defined as follows: A) the number of resource availability; B) the level of power utilization by stakeholders; C) potential capacity and resources, including financial, physical and human resources, authority, political power, knowledge; D) the ability of the stakeholder to affect the formulation and implementation of the policy, and other stakeholders; E) determine a stakeholder's ability to affect or block the implementation of a policy to influence policy decisions.
- “The level of participation” refers to regular attendance/participation in sessions, allocation of physical and human resources to politics, mobilization of resources and organizational capacities to implement politics.

| Stakeholders | Knowledge of subject | Position | Interests | Resources | Power/  influence | Level of participation |
| --- | --- | --- | --- | --- | --- | --- |
| The Parliament of Iran |  |  |  |  |  |  |
| National Headquarters for Coronavirus Control |  |  |  |  |  |  |
| Ministry of Health |  |  |  |  |  |  |
| Islamic Republic of Iran Army |  |  |  |  |  |  |
| Islamic Revolutionary Guard Corps |  |  |  |  |  |  |
| Disciplinary Command of the Islamic Republic of Iran |  |  |  |  |  |  |
| Iranian Traffic Police |  |  |  |  |  |  |
| Islamic Republic of Iran Broadcasting |  |  |  |  |  |  |
| Municipality |  |  |  |  |  |  |
| Governor/ governor-general |  |  |  |  |  |  |
| Hajj and Pilgrimage Organization |  |  |  |  |  |  |
| Planning and Budget Organization |  |  |  |  |  |  |
| Ministry of Culture and Islamic Guidance |  |  |  |  |  |  |
| Ministry of Cultural Heritage, Handicrafts and Tourism |  |  |  |  |  |  |
| Ministry of Education |  |  |  |  |  |  |
| Ministry of Science, Research and Technology |  |  |  |  |  |  |
| Ministry of Economic Affairs and Finance |  |  |  |  |  |  |
| Ministry of Cooperatives, Labour, and Social Welfare |  |  |  |  |  |  |
| Organization of Agriculture |  |  |  |  |  |  |
| Ministry of Interior |  |  |  |  |  |  |
| Ministry of Industry, Mine & Trade |  |  |  |  |  |  |
| Ministry of Information and Communications Technology of Iran |  |  |  |  |  |  |
| Ministry of Sport and Youth |  |  |  |  |  |  |
| Public transportation system (air, land and sea) |  |  |  |  |  |  |
| Central Bank of the Islamic Republic of Iran |  |  |  |  |  |  |
| National Development Fund of Iran |  |  |  |  |  |  |
| Social Security Organization |  |  |  |  |  |  |
| Organization for Combating Smuggling of Goods and Currency (Medication & Vaccine) |  |  |  |  |  |  |
| Judiciary |  |  |  |  |  |  |
| Trustees of the Holy Astans |  |  |  |  |  |  |
| Friday Prayer Policy Council |  |  |  |  |  |  |
| Islamic Development Coordination Council |  |  |  |  |  |  |
| Pharmaceutical companies |  |  |  |  |  |  |
| Drugs and medical equipment importing companies |  |  |  |  |  |  |
| Vaccine development companies |  |  |  |  |  |  |
| Basic and Complementary insurance |  |  |  |  |  |  |
| Private hospitals |  |  |  |  |  |  |
